# Transcriptional Responses of Resistant and Susceptible Fish Clones to the Bacterial Pathogen *Flavobacterium psychrophilum*

**DOI:** 10.1371/journal.pone.0039126

**Published:** 2012-06-13

**Authors:** Christelle Langevin, Mar Blanco, Samuel A. M. Martin, Luc Jouneau, Jean-Francois Bernardet, Armel Houel, Aurélie Lunazzi, Eric Duchaud, Christian Michel, Edwige Quillet, Pierre Boudinot

**Affiliations:** 1 INRA, Molecular Virology and Immunology, Domaine de Vilvert, Jouy en Josas, France; 2 Department of Animal Health, Faculty of Veterinary Sciences, Complutense University, Madrid, Spain; 3 Institute of Biological and Environmental Sciences, University of Aberdeen, Aberdeen, United Kingdom; 4 INRA, UMR1313 Génétique Animale et Biologie Intégrative, Domaine de Vilvert, Jouy en Josas, France; French National Centre for Scientific Research – Université Aix-Marseille, France

## Abstract

*Flavobacterium psychrophilum* is a bacterial species that represents one of the most important pathogens for aquaculture worldwide, especially for salmonids. To gain insights into the genetic basis of the natural resistance to *F. psychrophilum*, we selected homozygous clones of rainbow trout with contrasted susceptibility to the infection. We compared the transcriptional response to the bacteria in the pronephros of a susceptible and a resistant line by micro-array analysis five days after infection. While the basal transcriptome of healthy fish was significantly different in the resistant and susceptible lines, the transcriptome modifications induced by the bacteria involved essentially the same genes and pathways. The response to *F. psychrophilum* involved antimicrobial peptides, complement, and a number of enzymes and chemokines. The matrix metalloproteases *mmp9* and *mmp13* were among the most highly induced genes in both genetic backgrounds. Key genes of both pro- and anti-inflammatory response such as IL1 and IL10, were up-regulated with a greater magnitude in susceptible animals where the bacterial load was also much higher. While higher resistance to *F. psychrophilum* does not seem to be based on extensive differences in the orientation of the immune response, several genes including complement C3 showed stronger induction in the resistant fish. They may be important for the variation of susceptibility to the infection.

## Introduction

The genus *Flavobacterium* belongs to the family *Flavobacteriaceae*, in the phylum *Bacteroidetes* [1]. *Flavobacterium* strains are Gram-negative, non spore-forming, strictly aerobic rods and are usually motile by gliding. They occur in a variety of environments and are especially common in freshwater habitats. Three *Flavobacterium* species, namely *F. columnare*, *F. branchiophilum* and *F. psychrophilum*, are pathogenic to fish [2].

*F. psychrophilum* is primarily a salmonid pathogen, though cases have occasionally been reported from non-salmonid fish [3]. Originally restricted to the United States and Canada, infections by *F. psychrophilum* first appeared in Europe during the mid-eighties [4] and were progressively reported from all major areas of salmonid aquaculture in both Northern and Southern hemispheres over the next decade. The distribution of the pathogen is now considered worldwide and the losses it causes to the salmonid industry are considerable. Outbreaks typically occur when water temperature is below 15°C.

Infection by *F. psychrophilum* may result in different pathological entities depending on the fish species, developmental stage, and geographical area [5], [6]. In the so-called “peduncle disease” and “bacterial cold-water disease”, ulcerative lesions occur in the area surrounding the adipose fin and progressively extend to the whole caudal peduncle [5]. Gill lesions and nervous forms of the disease were also reported [7], [8]. In Europe, *F. psychrophilum* infection mostly manifests itself as a septicemic form with high mortality known as the “rainbow trout fry syndrome” as it usually concerns rainbow trout fry and fingerlings [2], [6]. Specific clinical signs may be either absent or consist of ulcerative lesions associated with severe splenic hypertrophy [9]–[11].

The various experimental infection and challenge models that have been proposed (reviewed in [6]), using injection of/immersion in bacterial suspensions or cohabitation with diseased fish, have met with varying degrees of success. Good results have been obtained for *F. psychrophilum* in rainbow trout using subcutaneous, intramuscular, or intraperitoneal injection [12]. In spite of extensive research and publication of promising results obtained under experimental conditions [13], [14], [26], no vaccine is commercially available at the present time.

The virulence mechanisms of *F. psychrophilum*, especially at the molecular level, are yet to be fully elucidated. Many mechanisms potentially involved in virulence have been reported under experimental conditions, but their actual role during the course of natural disease has not been fully evaluated. The following factors may be particularly significant: the adherence to the fish egg, gill tissue and body surface [15]–[17], the production of various extracellular proteases [18]–[24], the iron acquisition mechanisms [25] and the resistance to the action of the complement present in the serum of rainbow trout [26]. Other important characteristics of *F. psychrophilum* may influence its transmission, such as ability to form biofilms [27], presence of asymptomatic carriers in rivers and fish farms [28], and vertical transmission of the pathogen through intra-ovum infection [29], [30].

Significant progress has been made elucidating many of the genes relevant to the salmonid immune system and these gene sequences provide tools for studying the teleost immune response to pathogens and vaccines. The study of the modifications in the expression of mRNAs for important cytokines, sensors and effector genes by infection is an important step to better understand fish immunity to pathogens and to further dissect the function of these genes *in vivo* [31]. Responses to other major bacterial pathogens of salmonid fish have been examined by transcriptome profiling, including *Aeromonas salmonicida* [32], *Yersinia ruckeri* [33], *F. psychrophilum* [34] and *Piscirickettsia salmonis* [35] which together have indicated mechanisms by which fish attempt to control the invading pathogen. Also, it was reported that spleen size is a good indicator of the rainbow trout resistance to *F. psychrophilum* [36]. However no studies to date have examined differences to the pathogen response by use of clonal lines of fish exhibiting susceptibility or resistance based on genetic background of the fish.

In the present study, we identified resistant and susceptible animals among a previously established collection of rainbow trout homozygous clonal lines [37]. We evaluated the host response to *F. psychrophilum* infection and studied the differences between resistant and susceptible fish. Anterior kidney (termed “pronephros” thereafter) was analysed as it is an important target of the infection, with high bacterial load. Fish were sampled at day 5 post-infection and the pronephros RNAs were analyzed using a 44 K rainbow trout micro-array. Specific expression profiling of selected relevant genes was also assessed with real time reverse transcriptase quantitative PCR (QPCR). We found that infection by *F. psychrophilum* induced strong modifications of the pronephros transcriptome in both resistant and susceptible lines, but only a small fraction of the differentially expressed genes showed line-specific responses. These results provide clues for the understanding of the differences of responses in resistant and susceptible fish and for the identification of the genetic basis of predisposition to this disease.

## Materials and Methods

### Ethics Statement

All animals were handled in strict accordance with good animal practice as defined by the European Union guidelines for the handling of laboratory animals (http://ec.europa.eu/environment/chemicals/lab_animals/home_en.htm) and by the Regional Paris South Ethics committee, and all animal work was approved by the Direction of the Veterinary Services of Versailles (authorization number 78-28).

#### Fish

Rainbow trout clonal lines originated from the INRA ‘synthetic’ strain (Sy). Homozygous, genetically uniform clonal lines were settled after two generations of gynogenesis (all maternal inheritance) and further propagated by within-line single pair mating between a female and a sex-reversed male as described in [31]. A set of clonal lines was screened for response to infection with *F. psychrophilum* and a range of susceptibility was observed (Quillet et al, in preparation). Two clonal lines exhibiting consistent difference in susceptibility after intramuscular injection of the JIP 02/86 strain of *F. psychrophilum* were selected from the whole set of rainbow trout lines for the present study. A3 (A3_r) was chosen as a ‘resistant’ line and B57 (B57_s) as a ‘susceptible’ line respectively.

#### Bacterial strain, infection protocol and assessment of bacterial load in tissues

Fish of the clonal lines A3_r and B57_s, hatched in the IERP INRA facilities, were reared in recirculated units supplied with 10°C dechlorinated tap water until the time of experimentation, when their weight was 120–150 g. For infection, the virulent *F. psychrophilum* strain JIP 02/86, cultured for 24 hours according to standard procedures, was used. In addition to well-preserved and fairly controlled virulent properties, the complete genome of this strain has been sequenced and is available [38]. Ten fish of each line were sampled, anaesthetized in phenoxyethanol (0.3 ml/l) and injected intra-muscularly with 2.6×10^4^ CFU of a 24 hours bacterial culture. Fish were injected on the side, close to the dorsal fin, and the bacteria were delivered around 2 mm deep in the muscle. Parenteral route was preferred to ensure that all individuals received approximately the same dose of bacteria, since waterborne infection with *F. psychrophilum* is poorly controlled and would have led to great variation in fish exposure to the pathogen. Ten fish were mock-infected in parallel with 0.1 ml physiological saline. After injection, infected and mock-infected control fish were kept in dedicated flow-through aquaria (10°C) until subsequent sacrifice and sampling. Pronephros samples were kept in RNA later (Quiagen) until RNA preparation for Microarray hybridization (7 samples per condition) or for QPCR analysis (3 samples per condition).

### Micro-array slides and hybridizations

Micro-array experiments were performed using an Agilent-based micro-array platform with 4×44 K probes per slide (Agilent Design ID: 016320). This platform is a rainbow trout resource designed by Salem *et al*. [39]. The array was based on oligonucleotides designed by an assembly of rainbow trout ESTs performed by TIGR. The micro-array gene annotations have been reanalysed by the Sigenae team (INRA, Toulouse, France; http://www.sigenae.org/).

For micro-array analysis, we examined four RNA preparations from *F. psychrophilum*-infected fish and from control fish for each genetic background (biological replicates). Each sample comprised an equal quantity of RNA. RNA stability and integrity was assessed using Bioanalyzer (Agilent), and only RNA with RIN>8 were used. Each experimental sample was Cy3-labeled following the procedure recommended by Agilent, and was hybridized in one-color experiment. Briefly, the RNA was first reverse transcribed, using a poly(dT)-T7 primer. The Cy3 was then incorporated by a T7 polymerase mediated transcription, and the excess of dye removed using the RNeasy kit (Quiagen). The level of dye incorporation was assessed by spectrophotometry (Nanodrop ND1000, LabTech). Hybridizations were performed in a rotisserie-style Micro-array Hybridization Oven (Agilent) overnight (18 h) at 65°C. Following hybridization, the slides were rinsed in gene expression wash buffers 1 and 2 (Agilent) following the manufacturer's instructions. The slides were scanned using an Agilent high-resolution micro-array scanner at a resolution of 5 µm. Images, saved as *.TIF files, were extracted, and initial analysis were performed with Feature Extraction software v9.5.3 (Agilent). At least four individuals from each condition (i.e. 4 control B57_s control, 5 infected B57_s, 6 control A3_r an 7 infected A3_r) were used for the micro-array hybridization and analysis. The data discussed in this publication have been deposited in NCBI's Gene Expression Omnibus and are accessible through GEO Series accession number GSE35448.

### Micro-array data analysis

Statistical analysis was performed using R software (http://www.r-project.org/). Scanner output files were imported using read.maimages, a function of the limma package. For probes present more than once on the micro-array, the average of signals was considered. A within-normalization by subtraction of the median signal value was operated for each micro-array. Limma functions were used to perform the differential expression analysis. Raw pValues have been adjusted using Benjamini-Hochberg procedure [40]. The number of individuals analyzed for each condition was unequal (varying from 4 to 7), but at least four individuals were used per condition, and the statistical procedure was valid in these conditions.

### Ingenuity pathway analysis

The Ingenuity pathway analysis software (IPA) tool was used to perform pathway analysis and to search for functional networks involving the genes modulated by the infection. IPA consists of a unique curate knowledge base of cellular interactions and regulatory events mined from the peer-reviewed literature. Since the IPA system supports only human, rat and mouse, we searched for the best human counterparts of the trout genes identified by our micro-array analysis and used these converted datasets to query the ingenuity base. The default values of IPA parameters were used for our analyses. The following procedure was followed to extract tentative lists of human counterparts of relevant trout sequences. A first relationship of homology had been previously established between probes and public trout ESTs using the SigReannot pipeline [41]. We therefore used these ESTs to identify the most similar sequence from Swissprot, RefSeq RNA or RefSeq Protein databases. These sequences were then extracted and used as baits for blastx queries to the human uniprot fasta library. The best hit was kept for IPA analysis.

### GO annotation

As mentioned above, micro-array probes were linked with Swissprot or Refseq entries when possible. GO and Kegg annotations have been extracted from Swissprot database for each of these Swissprot accessions. For Refseq annotation, accession IDs were submitted to DAVID gene ID conversion tool [42], [43]. From 224 IDs, 151 could be successfully converted to a DAVID identifier, allowing to retrieve a functional annotation and to extract GO and Kegg annotation.

### Quantitative PCR

Trout pronephros were kept in RNA later (Quiagen) until RNA extraction. After tissue homogenization with ceramic beads (1–1.2 mm; N1100; Mineralex, France) and two rounds of centrifugation (6000 *g*; 15 s; Precyllis 24), RNA was prepared using Trizol reagent. The RNA pellet was washed with cold 70% ethanol, air-dried and redissolved in 350 µl RLT buffer. RNA was then subjected to an on-column DNase treatment using the RNeasy Mini kit (Qiagen). RNA concentrations were measured by spectrophotometry (Nanodrop, Thermo Scientific). RNA stability and integrity was assessed using Bioanalyzer (Agilent), and only RNA with RIN>8 were used. Reverse transcription reactions were as follows: 2.5 µg RNA, 2 µl 10 mM poly (dT)25 primers and 1 µl 10 mM dNTP mix were incubated for 5 min at 65°C, followed by 1 min incubation on ice. Four µl 5× first strand buffer, 2 µl 0.1 M DTT, 1 µl RNase inhibitor and 1 µl Superscript II Reverse Transcriptase (RT, Invitrogen) were added to each sample, and the reaction was incubated for 50 min at 42°C, followed by an enzyme-inactivation step of 15 min at 70°C.

For QPCR, 5 µl cDNA, 5 µl mix of forward and reverse primers (300 nM each) were added to 10 µl Power SYBR® Green Master mix (AB Gene). QPCR (10 min at 95°C, 40 cycles of 15 s at 95°C and 60 s at 60°C) was carried out with the Mastercycler® Realplex (Eppendorf). After each run, melting curves were produced by detecting fluorescence from 60 to 95°C at 1°C intervals. ELF-1α gene expression served as an internal standard. Fold changes following stimulation were calculated with the Pfaffl method [44], as a ratio of target gene vs. reference gene relative to expression in unstimulated control samples. Datasets were then analyzed using QBAse (Biogazelle). All primer sequences are shown in **Table 1** **.**

**Table 1:** Primer sequences used in the QPCR analysis.

Gene	Primers	Sequences
**TNF α-1**	TNF-α1 Fwd	AGCATGGAAGACCGTCAACGAT
	TNF-α1 Rev	ACCCTCTAAATGGATGGCTGCTT
**TNF α-2**	TNF-α2 Fwd	GGAGGCTGTGTGGCGTTCT
	TNF-α2 Rev	TGCTGACACCAGGCAAAGAG
**IL-1β-1**	IL-1β1 Fwd	GGAGAGGTTAAAGGGTGGCGA
	IL-1β1 Rev	TGCCGACTCCAACTCCAACA
**IL-6**	IL-6 Fwd	ACTCCCCTCTGTCACACACC
	IL-6 Rev	GGCAGACAGGTCCTCCACTA
**TLR 2**	TLR2 Fwd-1	TCCTGCGTCTATGTCTGCAC
	TLR2 Rev-1	CTCCAGGGAGCACCAGTTAC
**TLR 22**	TLR22 Fwd	TGGACAATGACGCTCTTTTACC
	TLR22/22L Rev	GAGCTGATGGTTGCAATGAGG
**TLR5**	TLR5 Fwd	GGCATCAGCCTGTTGAATTT
	TLR5 Rev	ATGAAGAGCGAGAGCCTCAG
**TLR3**	TLR3 Fwd	AGCCCTTTGCTGCCTTACAGAG
	TLR3 Rev	GTCTTCAGGTCATTTTTGGACACG
**TLR9**	TLR9 Fwd1	TGGATGAAAAGGTGGACGTGGC
	TLR9 Rev1	GGCCAGGACAGAACAAACTT
**IL-2**	IL-2 Fwd	CATGTCCAGATTCAGTCTTCTATACACC
	IL-2 Rev	GAAGTGTCCGTTGTGCTGTTCTC
**IL-10**	IL-10 Fwd	CGACTTTAAATCTCCCATCGAC
	IL-10 Rev	GCATTGGACGATCTCTTTCTTC
**IFNγ**	γIFN Fwd	GCTGTTCAACGGAAACCCTGTTT
	γIFN Rev	GTCCAGAACCACACTCATCAA
**mmp9**	mmp9a_F	TGTGTCCGTCACGTTCCCTGG
	mmp9a_R	CCGCAGCGAGGTGCCTTCAT
**mmp13**	mmp13_F	GCACCTTCTCTCTGCCCCGCAG
	mmp13_R	GCTCTGTTGTGGTTTGCTGC
**IL-11**	IL11_F	TCAACTCCCTTGAGATGAGACC
	IL11_R	TCCTGGGAAGACTGTAACACATC
**IFN1 (short)**	IFNc_FT	GCGAAACAAACTGCTATTTACAATGTATA
	IFNc_RT	TCACAGCAATGACACACGCTC
**ELF-1α**	ELF1α Fwd	CAAGGATATCCGTCGTGGCA
	ELF1αRev	ACAGCGAAACGACCAAGAGG
***F. psychrophilum***	Fp16s F	GAGTTGGCATCAACACAC
**16 S RNA**	Fp16s R	TCCGTGTCTCAGTACCAG

## Results

### Rainbow trout lines resistant and susceptible to *F. psychrophilum* infection

**Figure 1:**
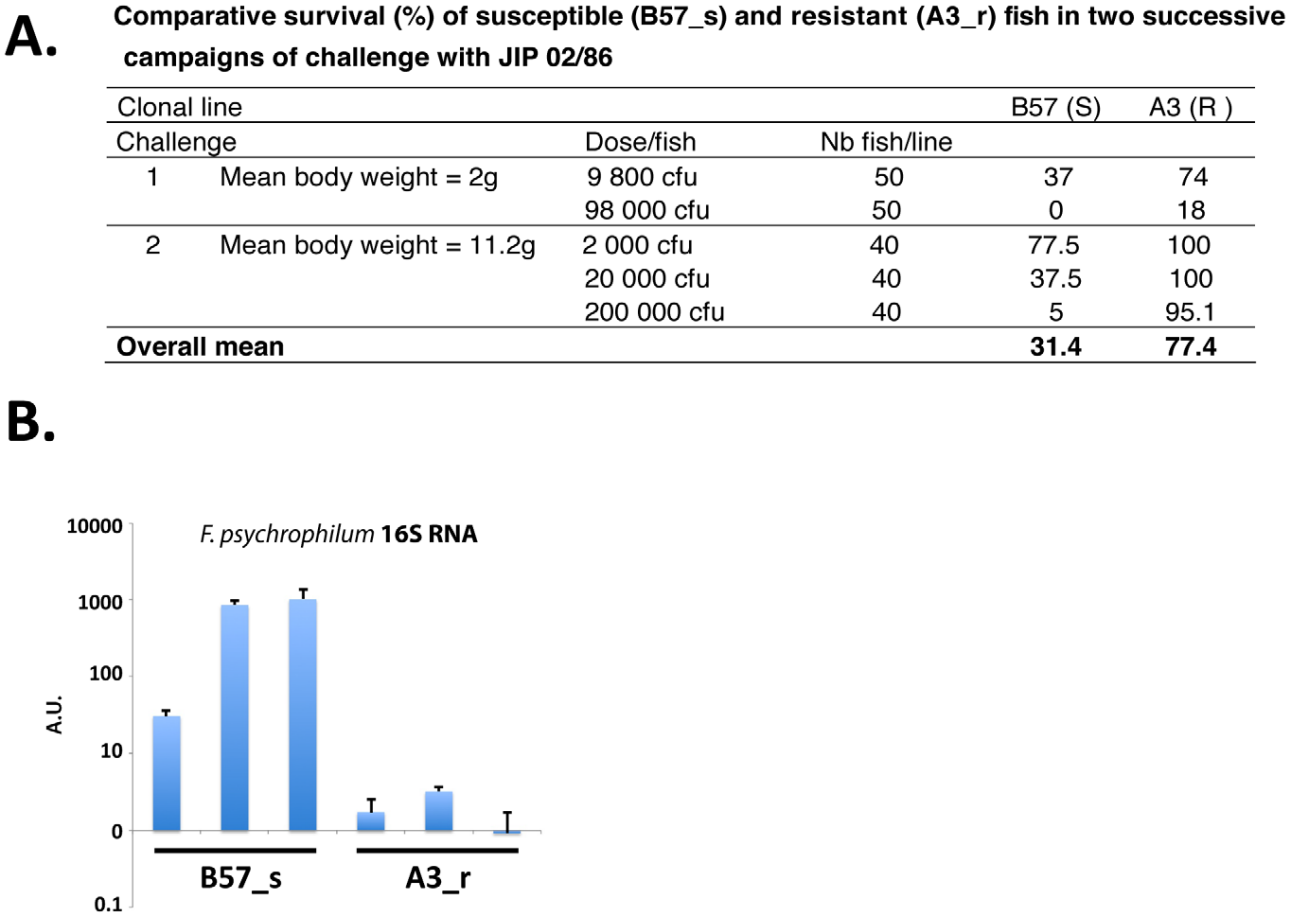
Rainbow trout clonal lines resistant and susceptible to *F. psychrophilum*. A) Comparative survival (%) of A3_r (resistant) and B57_s (susceptible) lines after infection with the JIP 02/86 strain of *F. psychrophilum*. Fish were challenged by intramuscular injection at different sizes and infectious doses. Survival was assessed when the mortality had stabilized (on day 32 for challenge 1 and on day 40 for challenge 2. Individuals used in the present study were the control sibs of challenge 2. B) Assessment of the bacterial load in the pronephros from the infected resistant and susceptible fish that were used for QPCR presented on figure 4. The 16 S RNA was quantified using a real time QPCR validated for diagnostic of *F. psychrophilum*. Consistent results were observed from the fish used for micro array hybridization when tested (B57_s, n = 4, bacterial load 97 to 3372; A3_r, n = 3, bacterial load 0.003 to 6).

Clonal lines A3 and B57 were chosen among a set of clonal lines that were repeatedly challenged against the bacterium (Quillet et al, in preparation). The clonal lines A3 and B57 consistently exhibited contrasted resistance (**Figure 1A**), and were chosen as ‘resistant’ and ‘susceptible’ lines, respectively and named hereafter A3_r and B57_s. Control sibs of challenge 2 (**Figure 1A**) were used for transcriptome analysis at a later developmental stage, which possesses both innate and adaptive components of immunity.

To produce a first general description of the host response to the infection by *F. psychrophilum* in resistant and susceptible fish, we performed comparative micro-array analyses of the pronephros transcriptome from infected and control trouts with resistant (A3_r) versus susceptible (B57_s) background. The pronephros was chosen as a key lymphoid and hematopoietic tissue and a target of the *F. psychrophilum* infection. The impact of the infection on the transcriptome was studied five days after infection in order to analyze a fully developed response, whilst before the onset of clinical disease and the physiological stress due to lesions. The bacterial load in the pronephros five days post infection was significantly higher in susceptible compared to resistant fish, as shown on figure 1B. Since *F. psychrophilum* 16 S RNA was quantified from the individual samples, it provided a direct assessment of the bacterial load in each animal. No specific signal was detected in control fish. Additionally, *F. psychrophilum* could be re-isolated from pronephros samples of B57_s infected fish, but not from the A3_r.

### The core response of both resistant and susceptible clonal lines is a typical innate response to bacteria

**Figure 2:**
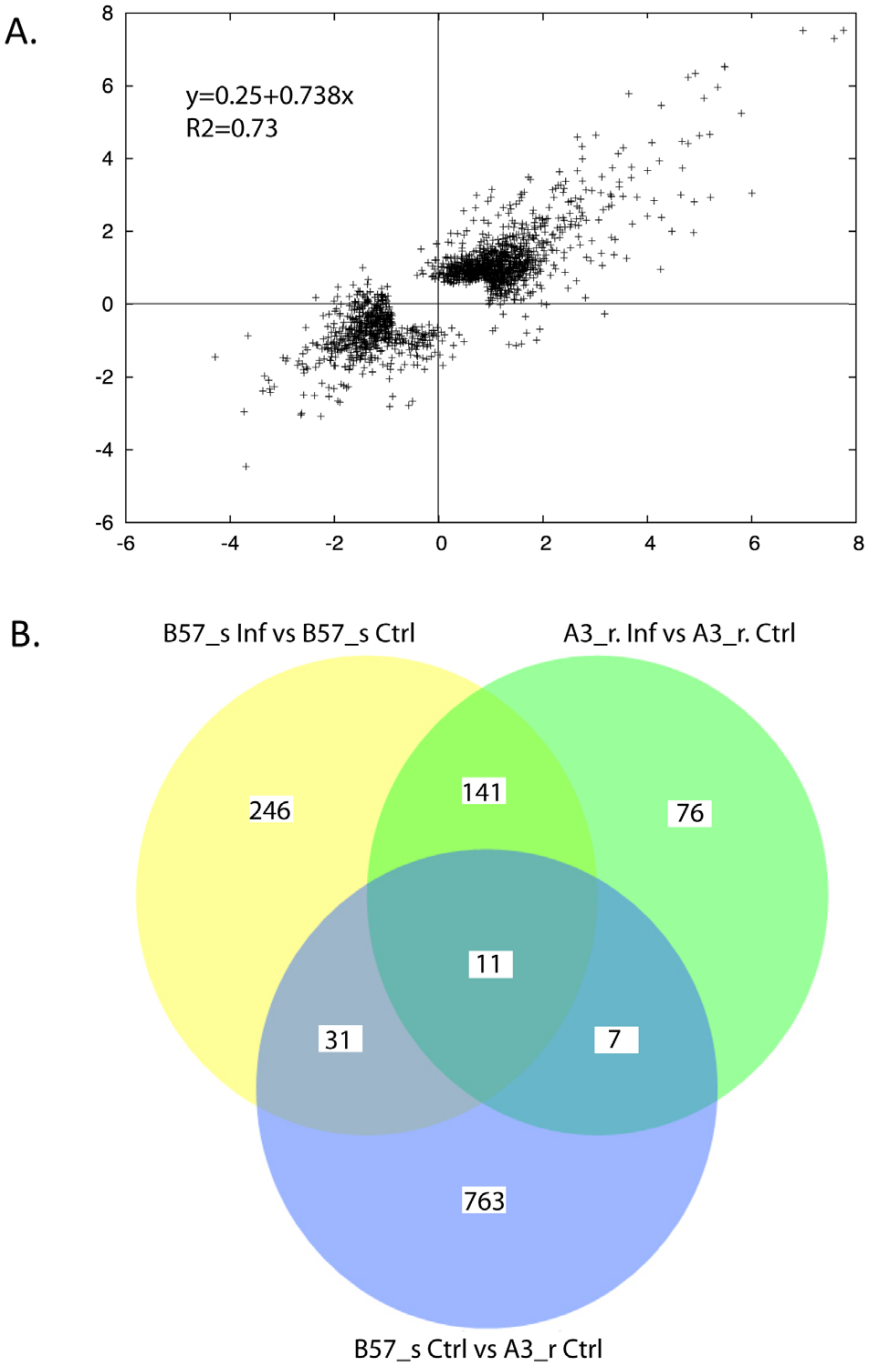
Global transcriptome response of fish pronephros to *F. psychrophilum* infection. A) LogFoldChange/LogFoldChange representation of all probes significantly modulated by *F. psychrophilum* infection in the pronephros of susceptible (x) and resistant (y) clonal lines. All adjusted *p* value (apv)<1% in both resistant and susceptible fish, no constraint on fold change. The distribution centered on a diagonal axis indicates that fold changes are globally correlated in resistant and susceptible clones. Parameters of the linear regression are indicated. B) Venn diagram of probes providing differential signals (apv<1%; FC>3 or FC<0.33) between infected or control susceptible fish (B57_s. Inf. versus B57_s. Ctrl.), between infected or control resistant fish (A3_r Inf. versus A3_r Ctrl.), and between susceptible and resistant control fish (A3_r Ctrl. versus B57_s Ctrl.).

The modifications of the pronephros transcriptome following infection were analyzed in resistant (A3_r) and susceptible (B7_s) fish in comparison with control fish of each group. Overall, fold change values (FC) appeared well correlated in the two clonal lines (**Figure 2A**), indicating that their response to the bacterial infection was globally similar, slightly higher in the susceptible animals. A common core response was observed: 130 probes were up-regulated (adjusted *p* value (apv)<1%; FC>3) and 22 probes down-regulated (apv<1%; FC<0.33) in both clonal lines, generally with comparable fold change values (**Figure S1**). However, more probes were found significantly differential (apv<1%; FC>3 or FC<0.33) in the susceptible than in the resistant fish (**Figure 2B**), suggesting that the response may involve more genes in the context with the higher bacterial load.

The genes up-regulated following bacterial infection are indicative of a strong response targeting mainly matrix metalloproteases, cell activation signaling, and secreted soluble factors involved in immunity (**Table 2**). The complete list of modulated genes is provided in **Figure S1**.

**Table 2:** Immunity related genes up-regulated in rainbow trout clonal lines B57_s and A3_r following *F. psychrophilum* JIP 02/86 infection.

ID	Fold change		Tentative Annotation	Adj. p value	
	B57_s	A3_r		B57_s	A3_r
**Kinases and phosphatases**					
TC125014	**217,0**	**184,0**	Tyrosine-protein kinase Lyn	2,69E-11	1,58E-07
TC124742	**191,6**	**157,4**	Tyrosine-protein kinase Lyn	3,19E-11	1,58E-07
**AMP**					
TC108552	**55,8**	**38,0**	cathelicidin antimicrobial peptide	1,81E-06	1,02E-03
TC95594	**11,6**	**19,7**	cathelicidin antimicrobial peptide	4,14E-08	1,97E-04
TC119632	**10,9**	**17,6**	cathelicidin antimicrobial peptide	4,95E-08	2,22E-04
TC116498	**13,9**	**7,7**	Hepcidin-1	1,89E-07	4,05E-03
**MMP, ubiquinone, prostaglandin reductase, arginase, …**					
TC131371	**44.54**	**92.61**	NADH dehydrogenase [ubiquinone] 1A5	1,39E-06	9,24E-06
TC115587	**44.74**	**91.13**	NADH dehydrogenase [ubiquinone] 1A5	1,51E-06	7,25E-06
TC127722	**25.48**	**13.37**	Metalloreductase STEAP4	2,12E-08	1,40E-04
TC95675	**4.89**	**9.64**	Matrix metalloproteinase-9	4,33E-06	3,32E-05
TC100961	**4.54**	**8.83**	Matrix metalloproteinase-9	1,52E-05	5,90E-05
TC121902	**5.22**	**8.39**	Matrix metalloproteinase-9	6,07E-06	2,70E-04
TC99244	**64.16**	**8.28**	Matrix metalloproteinase-9	5,14E-09	2,02E-04
TC112624	**9.91**	**7.78**	Metalloreductase STEAP4	3,69E-07	1,14E-04
TC105973	**5.23**	**5.19**	Prostaglandin reductase 1	7,41E-07	5,79E-07
TC95147	**4.9**	**5.16**	Prostaglandin reductase 1	2,22E-06	4,69E-07
TC116823	**4.17**	**5.05**	transferrin receptor 1b-like	2,20E-04	4,75E-05
TC118982	**5.8**	**4.55**	Tripartite motif-containing protein 7	6,54E-05	9,73E-03
TC130649	**3.59**	**4.27**	NADH dehydrogenase [ubiquinone] 1A5	7,61E-03	3,58E-04
TC97442	**6.58**	**5.12**	Arginase-2	1,76E-07	3,79E-06
**Cytokines & Receptors**					
TC118120	**25.34**	**22.22**	Thymosin	1,86E-08	5,17E-05
TC122812	**27.51**	**21.31**	C-C motif chemokine 21	1,08E-08	2,96E-05
TC115195	**5.05**	**9.67**	Interleukin-8	3,85E-04	7,36E-03
TC110491	**7.08**	**8.8**	CC chemokine with stalk CK2	1,45E-06	4,45E-05
TC125695	**6.59**	**7.4**	CC chemokine with stalk CK2	1,39E-06	2,01E-04
TC118249	**29.78**	**7.03**	CC chemokine	3,14E-07	6,63E-03
TC122848	**3.02**	**4.27**	Chemokine receptor-like 1	2,29E-03	1,99E-04
TC102213	**3.29**	**3.4**	Leukocyte cell-derived chemotaxin-2	2,79E-05	3,58E-04
TC95482	**24.94**	**8,0**	Interleukin1 beta	4,76E-06	***1,84E-02***
TC132235	**22.15**	**3.99**	SOCS3	1,08E-07	***9,00E-02***
**IFN**					
TC105751	**6.23**	**5.08**	IFITM3	2,18E-06	2,24E-04
**Acute phase proteins**					
TC131580	**30.27**	**81.08**	Serum amyloid A-5	6,99E-06	1,90E-05
TC121913	**27.51**	**75.11**	Serum amyloid A-5	4,85E-06	6,15E-05
TC128889	**12.5**	**55.08**	Serum amyloid A-1	1,04E-04	3,41E-05
TC121607	**11.56**	**7.8**	lysozyme II	4,82E-06	7,78E-04
TC121304	**3.87**	**4.42**	Serum amyloid A	2,62E-03	3,46E-04
**Cell surface receptors**					
TC110053	**18.74**	**15.24**	Low affinity Ig epsilon Fc receptor	4,40E-09	3,12E-06
TC102195	**11.01**	**9.33**	CD209	1,33E-05	8,24E-04
TC113535	**5.78**	**7.14**	MUC18	1,90E-06	9,24E-06
TC103340	**5.81**	**7.06**	MUC18	2,30E-06	2,42E-05
TC114388	**9.96**	**6.57**	Low affinity Ig epsilon Fc receptor	4,10E-08	9,08E-05
**Complement**					
TC119105	**4.97**	**12.48**	Complement factor H-related protein 1	2,89E-04	3,17E-04
TC130908	**5.41**	**10.4**	Complement C3	2,30E-06	7,16E-04
TC101299	**7.66**	**8.71**	Complement C8	2,22E-06	9,26E-05
TC105891	**3.11**	**8.44**	Mannose-binding protein	1,75E-03	7,52E-04
TC108922	**3.02**	**8.11**	Mannose-binding protein	2,46E-03	1,02E-03
TC99951	**6.16**	**7.52**	Complement component C8	3,21E-06	1,24E-04
TC103014	**5.6**	**6.99**	Complement component C8	3,70E-06	1,72E-04
TC125288	**6.77**	**5.37**	Complement component C7	2,32E-06	4,96E-04
TC130006	**3.98**	**4.98**	Complement C3	5,85E-04	5,90E-05
TC124643	**5.35**	**4.32**	Complement factor H	1,24E-04	7,06E-03
TC109163	**3.58**	**4.13**	CD59	3,03E-03	8,95E-03
TC97935	**6.7**	**5.74**	Complement factor H	2,90E-05	1,88E-03

Genes of immunity induced in both resistant (A3_r) & susceptible (B57_s) fish (FC>3 & adj p val<1%).

### Matrix proteins and cell activation signaling

Matrix metalloproteases mmp9 (that is typically induced downstream TLR signaling) and mmp13 were significantly up-regulated in both clonal lines. *mmp9* was in fact represented on the micro-array by four probes corresponding to different TIGR contigs. Multiple alignments of all these mmp9-related contigs indicate that they constitute divergent 3′ UTR of transcripts encoding mmp9-like proteins with similar but non-identical C- terminus. It was therefore not possible to determine whether the different probes correspond to different *mmp9* genes or to different splicing variants of a unique gene. All the probes indicate that *mmp9* is up-regulated after infection. STEAP4, a gene encoding a plasma membrane metallo-reductase involved in the transport of iron and in the control of inflammatory cytokines [45] was also highly induced by the infection. The immuno-regulatory kinase gene *lyn*, represented by two probes, showed the strongest induction in both clones (fold>150), indicating that the 5 days at which these fish were sampled represents a well-developed response to the pathogen. Indeed, LYN is a Src tyrosine kinase expressed in B lymphocytes and myeloid cells where it operates signal transduction from cell surface receptors that lack intrinsic tyrosine kinase activity, and has a general regulatory role on cell activation.

### Antimicrobial peptides, cytokines and other soluble factors

Two types of AMP (cathelicidins and hepcidins) were among the most strongly induced genes, confirming the importance of these effectors in the response to *F. psychrophilum*. Several chemokines including IL8-like molecules were also over-expressed after infection, suggesting that a re-allocation of immune cells occurred upon inflammation and *F. psychrophilum* infection. While the TNFα transcript was not clearly up-regulated, either in resistant or in susceptible fish, the key pro-inflammatory cytokine IL1β was strongly induced by the infection. IL1β appeared significantly more induced in the susceptible clone, thus following the bacterial load. Concerning the immuno-modulatory cytokines, the IL10 signaling was among the pathways apparently triggered by the infection: a SOCS3-like transcript and Arginase-2 were up-regulated, suggesting that the expression of IL10 itself could be affected. Finally, the complement system was also strongly modulated.

In parallel, a smaller gene subset was down-regulated following infection, and did not appear to be directly connected to the antibacterial or inflammatory response. Rather, a functional analysis performed with Ingenuity identified a connection with renal and urological diseases based on aquaporin, carbonic anhydrase (significantly modulated in both susceptible and resistant fish) as well as parvalbumin and tubulinb (significantly modulated in susceptible fish only). This connection likely represents the consequence of the pronephros tissue lesions induced by the bacteria.

### Resistant and susceptible clonal lines display subtle variations of inflammatory response after *F. psychrophilum* infection

To obtain greater insights in the differences of response between resistant and susceptible lines, we specifically searched for probes with contrasted hybridization patterns.

Only a few probes (n = 28) were significantly up- or down- regulated in one line (apv<1%; FC>3 or <0.33) while being not affected in the other one (*i.e.* 0.5<FC<2 with apv<5%) (**Table 3**). Only two genes following this pattern and potentially important for the antibacterial response were identified: a probe similar to the lysozyme CII, that was up-regulated only in the resistant clone A3_r and two probes targeting metalloprotease inhibitors that were down-regulated only in the resistant clone B57_s.

**Table 3:** Genes up- (fold change>3) or down-modulated (fold change<3) in rainbow trout clonal lines B57_s or A3_r following *F. psychrophilum* JIP 02/86 infection.

Modulated in B57_s but not in A3_r*						
ID	Fold change		Tentative Annotation		Adj. p value	
	B57_s	A3_r			B57_s	A3_r
**Induced**						
TC124901	3.9	1.9	Purine nucleoside phosphorylase	5.32E-05	2.31E-02	
TC100797	3.6	1.8			1.97E-05	2.57E-02
TC120803	3.4	1.9	Cysteine protease ATG4D	2.18E-03	2.28E-02	
TC104704	3.4	1.9			3.50E-04	4.68E-02
TC114814	3.2	1.8	macrophage myristoylated C kinase	2.96E-03	4.55E-02	
TC124219	3.1	1.9	Synaptic vesicle membrane protein VAT-1	3.06E-04	4.17E-02	
TC127444	3.1	2.0	Metalloreductase STEAP4	9.39E-03	7.93E-03	
TC127011	3.1	1.9			2.29E-03	2.04E-02
**Repressed**						
TC121666	0.3	0.5	Kunitz-type protease inhibitor 1 (spit1)	1.24E-04	3.21E-02	
TC102625	0.3	0.6			1.22E-04	2.46E-02
TC108326	0.3	0.6	Aminopeptidase		2.91E-04	4.23E-02
TC127298	0.3	0.5	Retinol dehydrogenase 3	7.58E-05	1.40E-03	
TC107654	0.3	0.6	Glycogen phosphorylase	3.54E-04	3.52E-02	
TC98081	0.2	0.5	Asialoglycoprotein receptor	1.20E-04	3.71E-02	
TC119260	0.2	0.5	Asialoglycoprotein receptor	9.85E-05	1.30E-02	
TC117957	0.1	0.5	CC motif chemokine	1.08E-08	4.60E-02	

Modulated in A3_r but not in B57_s *						
ID	Fold change		Tentative Annotation		Adj. p value	
	B57_s	A3_r			B57_s	A3_r
**induced**						
TC127723	1.9	7.1	Lysozyme C II		2.77E-02	8.38E-06
TC121169	1.8	4.9	Oncorhynchus mykiss toxin-1	2.21E-02	5.44E-03	
TC122221	1.9	4.7	Oncorhynchus mykiss toxin-1	9.95E-03	6.83E-03	
TC103288	1.9	3.7	E3 ubiquitin-protein ligase RNF144A-A	1.20E-02	2.83E-04	
TC126469	1.9	3.5	Glutathione peroxidase 2	1.48E-02	1.39E-04	
TC104470	1.8	3.4	Glutathione peroxidase 2	2.16E-02	1.49E-04	
TC95197	1.9	3.1	Glutathione peroxidase 2	1.24E-02	2.97E-04	
**Repressed**						
TC108609	0.5	0.3	GATS-like protein		1.63E-02	1.44E-03
TC100173	0.5	0.3	Metalloproteinase inhibitor 2	1.32E-02	2.35E-03	
TC96675	0.5	0.3	Metalloproteinase inhibitor 2	1.73E-02	8.59E-04	
TC109784	0.5	0.2	neurotoxin/C59/Ly-6-like protein-like	6.80E-03	5.92E-05	
TC101706	0.5	0.1	Cytosolic sulfotransferase 3	3.50E-02	2.07E-04	

* Contraints for modulated genes ((FC>3 or <0.3; adj.p<1%) and for non mudulated genes (0.5<FC<2; adj. p<5%).

were selected to ensure that the fold change of non modulated genes was close 1. Higher adjusted p value threshold (5%) was set up to keep as much relevant genes as possible.

A second set of probes with contrasted patterns consisted in probes for which up- or down- regulation was significant in only one of the two lines, while micro-arrays results showed either no modulation or a large variation of the expression level, in the other one (i.e. a high adj. *p* value, above the 0.05 threshold). Such a pattern revealed a differential behavior of the corresponding gene in resistant and susceptible fish following infection. This set comprised a significant number of probes (n = 193) (**Figure S2**). Interestingly, the IL1 receptor (IL1R2 but not IL1R1) belongs to this category and seemed to be induced with significant fold change and consistency in the susceptible background only. Among the cytokines represented on the micro-array, IL11 showed a similar pattern, being apparently induced in susceptible fish but not with a significant *p* value in the resistant ones. However, this pattern could be validated by QPCR (see below). A homolog of the lipopolysaccharide-induced tumor necrosis factor-alpha factor (LITAF) was also modulated with a comparable pattern. However, it is not known if this transcription factor regulates rainbow trout TNFα as human LITAF does. The trout TNFα was not found upregulated by the *F. psychrophilum* infection.

Finally, a last set of genes showing a different response in resistant and susceptible fish comprised genes that responded in both lines, but to a greater magnitude in a given genetic background compared to the other one. A clustering analysis classified these genes into four subsets.

**Figure 3:**
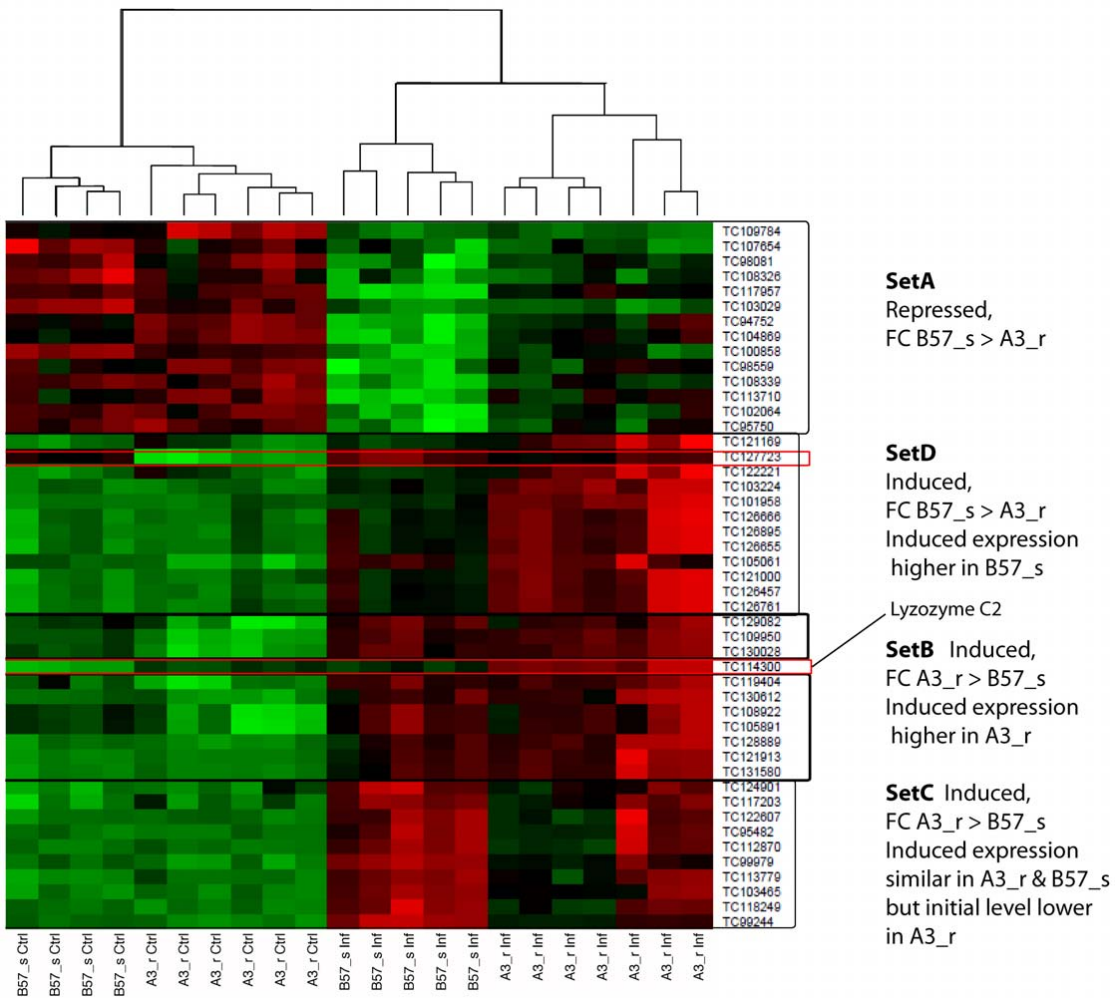
Clustering analysis of probes differentially modulated in resistant and susceptible clones. The clustering of probe signal identified gene subsets with specific expression patterns. Set A: genes repressed more in susceptible fish, compared to resistant. Set B: genes induced more in resistant than in susceptible fish, reaching higher expression level in resistant animals. Set C: genes induced more in resistant than in susceptible ones, reaching similar expression levels from lower basal expression before infection. Set D: genes more induced in susceptible than in resistant fish.

For thirteen genes, the decrease was at least twofold more in susceptible B57_s fish, compared to resistant ones (A3_r) (**Figure 3** **, Set A** and **Table 4**). Among these genes, a CC chemokine may be directly involved in the response to the bacteria.

**Table 4:** Different patterns of contrasted responses in rainbow trout clonal lines B57_s and A3_r following *F. psychrophilum* JIP 02/86 infection.

	ID	Fold change		Tentative Annotation	Adj. P value		*Relative repression
							or induction factor
		B57_s	A3_r		B57_s	A3_r	between clonal lines
**Set A**	**Fold Change B57_s≫ A3_r**						**Rel. Repression factor**
	TC95750	**0,20**	**0,46**		3,66E-05	1,78E-02	2,29
	TC102064	**0,20**	**0,46**		1,77E-05	9,21E-03	2,35
	TC113710	**0,17**	**0,42**	TBC1 domain family member 2A	2,06E-05	2,62E-02	2,40
	TC108339	**0,13**	**0,36**	Heme-binding protein 2	8,87E-07	3,30E-03	2,66
	TC98559	**0,13**	**0,34**	slc4a	1,29E-04	2,44E-02	2,58
	TC100858	**0,05**	**0,36**	C-C motif chemokine	1,41E-08	3,93E-03	7,11
	TC104869	**0,15**	**0,31**	cytochrome P450 1A	3,16E-06	2,26E-03	2,04
	TC94752	**0,10**	**0,23**	cytochrome P450 1A	5,08E-07	4,43E-03	2,25
	TC103029	**0,10**	**0,26**	Scavenger receptor class B	1,45E-07	7,00E-05	2,60
	TC117957	**0,08**	**0,55**	C-C motif chemokine	1,08E-08	4,60E-02	6,93
	TC108326	**0,32**	**0,64**	aminopeptidase	2,91E-04	4,23E-02	2,03
	TC98081	**0,23**	**0,53**	Asialoglycoprotein receptor	1,20E-04	3,71E-02	2,32
	TC107654	**0,27**	**0,58**	Glycogen phosphorylase	3,54E-04	3,52E-02	2,10
**Set B**	**Fold change A3_r≫ B57_s**						**Rel. Induction factor**
	TC126761	**2,93**	**7,12**	Complement C3	6,26E-04	9,04E-05	2,44
	TC126457	**2,79**	**6,75**	Complement C3	1,78E-03	7,09E-05	2,41
	TC121000	**2,61**	**6,16**	Complement C3	2,34E-03	9,25E-05	2,36
	TC105061	**2,58**	**6,62**	Glutaminyl-peptide cyclotransferase	1,42E-03	3,19E-05	2,57
	TC126655	**2,53**	**5,66**	Complement C3	5,46E-03	1,36E-04	2,23
	TC126895	**2,36**	**6,31**	Complement C3	6,78E-03	8,88E-05	2,68
	TC126666	**2,21**	**5,45**	Complement C3	1,20E-02	2,02E-04	2,47
	TC101958	**2,03**	**8,89**		5,98E-03	9,18E-06	4,39
	TC103224	**2,00**	**7,65**	Retinoid-binding protein	8,14E-03	5,33E-06	3,83
	TC122221	**1,93**	**4,67**	*Oncorhynchus mykiss* toxin-1	9,95E-03	6,83E-03	2,42
	TC121169	**1,82**	**4,93**	*Oncorhynchus mykiss* toxin-1	2,21E-02	5,44E-03	2,71
	TC114300	**6,72**	**20,15**		2,34E-06	3,48E-06	3,00
	TC127723	**1,92**	**7,10**	Lysozyme C II OS	2,77E-02	8,38E-06	3,70
**Set C**	**Fold change A3_r≫ B57_s; final expression level similar in both backgrounds**						**Rel. Induction factor**
	TC131580	**30,27**	**81,08**	Serum amyloid A-5	6,99E-06	1,90E-05	2,68
	TC121913	**27,51**	**75,11**	Serum amyloid A-5	4,85E-06	6,15E-05	2,73
	TC128889	**12,50**	**55,08**	Serum amyloid A-1	1,04E-04	3,41E-05	4,41
	TC105891	**3,11**	**8,44**	Mannose-binding protein C	1,75E-03	7,52E-04	2,71
	TC108922	**3,02**	**8,11**	Mannose-binding protein C	2,46E-03	1,02E-03	2,68
	TC130612	**8,07**	**24,99**	Differentially regulated trout protein 1	4,97E-05	2,32E-04	3,10
	TC119404	**6,31**	**24,15**	L-serine dehydratase/L-threonine deaminase	2,44E-04	2,61E-05	3,82
	TC130028	**3,29**	**11,26**		4,30E-04	9,77E-07	3,42
	TC109950	**3,38**	**10,75**		1,72E-04	6,29E-07	3,18
	TC129082	**3,01**	**7,64**		1,02E-03	5,06E-04	2,54
**Set D**	**Fold Change B57_s≫ A3_r**						**Rel. Induction factor**
	TC99244	**64,16**	**8,28**	MMP9	5,14E-09	2,02E-04	7,75
	TC118249	**29,78**	**7,03**	CC chemokine	3,14E-07	6,63E-03	4,24
	TC103465	**16,02**	**5,35**		4,95E-08	2,58E-03	2,99
	TC113779	**19,30**	**5,21**	Collectin-12	1,86E-08	8,77E-03	3,71
	TC99979	**6,55**	**3,10**		1,76E-07	1,21E-03	2,11
	TC112870	**37,05**	**7,64**		1,07E-07	3,78E-02	4,85
	TC95482	**24,94**	**8,01**	Interleukin-1 beta	4,76E-06	1,84E-02	3,11
	TC122607	**7,65**	**3,34**	Jagunal homolog 1-A (jgn1a)	4,26E-06	4,64E-02	2,29
	TC117203	**5,28**	**2,41**		4,10E-06	1,29E-02	2,19
	TC124901	**3,88**	**1,87**	Purine nucleoside phosphorylase	5,32E-05	2,31E-02	2,07

Four gene sets (A–D) were identified by hierarchical clustering as shown in Figure 3.

* The relative repression or induction factor between fish clonal lines was calculated as follows: rf = Max (Fold change B57_s, Fold change A3_r)/Min (Fold change B57_s, Fold change A3_r).

Eleven genes were more effectively induced in resistant than in susceptible fish, reaching higher expression rate in the resistant animals (**Figure 3** **Set B** and **Table 4**). C3, retinoic acid binding protein and *Oncorhynchus mykiss* toxin-1 were among these genes.

Ten genes were also induced with a greater magnitude in resistant fish than in susceptible ones, but reached similar expression levels from lower basal expression before infection. Such genes comprised Serum Amyloid A (SAA), Mannose Binding Protein (MBP) and several non-annotated ESTs (**Figure 3** **, Set C** and **Table 4**). Following a slightly different pattern, lysozyme CII and an unknown gene were also more induced in resistant, but did not reach after infection the basal level observed in susceptible fish (framed in red in **Figure 3**). In fact, the fold change value for lysozyme CII was 1.9 in the susceptible clonal line, and was therefore previously commented as a gene induced only in the resistant fish (see above). Such a pattern may be misleading concerning its effective impact, which is probably stronger in the susceptible B57_s background.

Finally, ten genes were induced to a greater magnitude in susceptible B57_s than in resistant A3_r fish, including *mmp9*, a CC chemokine and *IL1*β (**Figure 3D** and **Table 4**).

It is important to note that a number of genes involved in antibacterial immunity were more induced in resistant than in susceptible fish showing that the transcriptional response of immune genes – including effectors such as SAA – was not explained by a mere correlation to the bacterial load.

### QPCR expression profiles of selected genes confirm that resistant and susceptible clonal lines have differential adjustments of bacterial induced inflammatory response

To further validate the results of the micro-array analysis and to compare further the response to infection in lines A3_r and B57_s, the expression of selected transcripts was assessed by QPCR.

**Figure 4:**
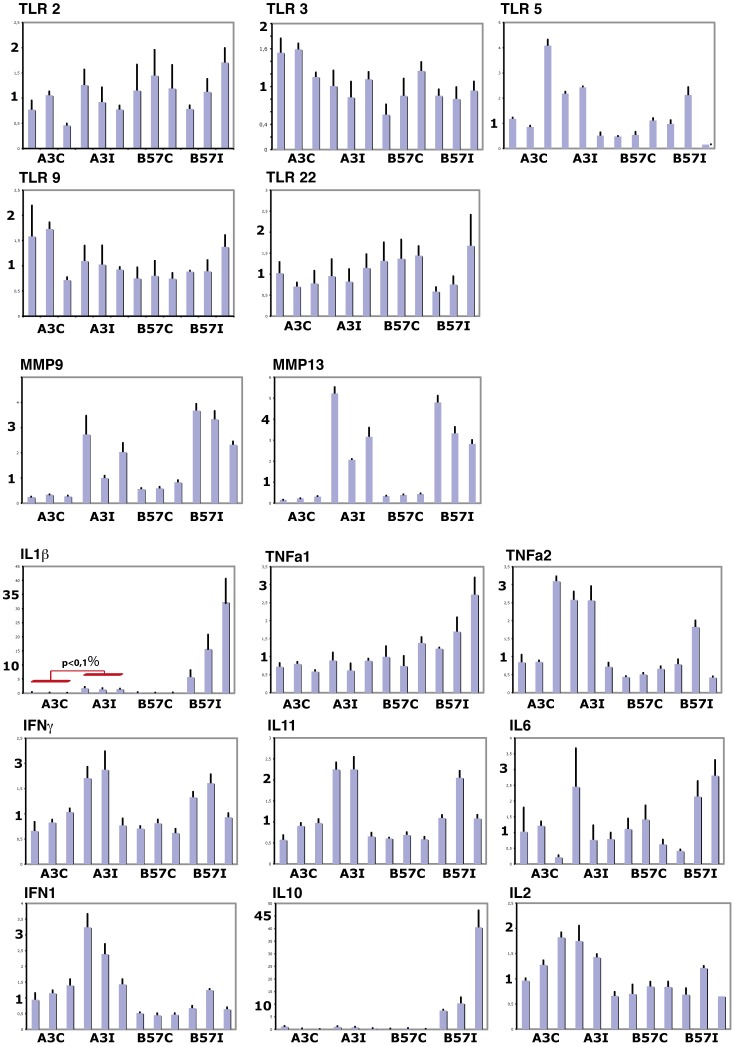
QPCR analysis of the impact of *F. psychrophilum* infection on the expression of selected genes. For each condition, gene expression was analyzed in the pronephros of three independent resistant A3-r or susceptible B57_s fish 5 days post inoculation. Each fish is represented as separate bars. The expression of a gene of interest in each individual is indicated as a ratio to the expression level of the house-keeping gene ELF-1α. Error bars represent the variation between technical triplicates.

No modulated TLR was identified by the micro-array analysis of the response to *F. psychrophilum* infection. To confirm this observation and to extend it to TLR that were not represented on the array, we assessed the expression of TLR2, 3, 5, 9 and 22 in the pronephros from resistant or susceptible, infected or control fish (i.e. from twelve animals). As shown in **Figure 4**, we observed a significant inter-individual variability (especially for TLR5) but none of these TLR was found to be induced by the infection, nor differentially expressed between the resistant and susceptible fish.

Concerning matrix proteins, the real time QPCR assay confirmed that the pronephric expression of both mmp9 and mmp13 was up-regulated 5 days post infection with *F. psychrophilum* in resistant as well as in susceptible fish.

To better understand the response induced by the bacterial infection, we also analyzed the modifications of key cytokines in resistant and susceptible animals. Typical pro inflammatory cytokines including IL1β, TNFα1, TNFα2, and IFNγ showed various expression profiles. IL1β (the prototype of inflammatory cytokines) was significantly induced in both resistant and susceptible fish, but considerably more in susceptible animals, which confirmed the trend observed in the micro-array analysis. The three other cytokines showed high inter-individual variability. These results were fully consistent with the micro-array analysis for IL1β. The other cytokines were not represented on the micro-array, and did not appear to be induced by the infection in QPCR experiments.

Among cytokines regulating the pro-inflammatory response, the up-regulation of IL11 was found significant only in susceptible B57_s fish by the micro-array analysis (Figure S2). Interestingly, QPCR confirmed this trend since IL11 appeared up-regulated in susceptible fish but only in two individuals among the resistant ones. In the third animal IL11 was expressed at the same level as in controls, while IL1β was consistently up-regulated in all individuals. Thus, IL11 QPCR experiments suggested a differential expression between infected and control susceptible fish, but with a lower ratio than expected from the micro-array analysis. The other anti-inflammatory cytokine IL10 was not represented on the micro-array; however, our analysis suggested an activation of IL10 signaling. We therefore quantified the IL10 transcript, and showed that IL10 was highly induced in susceptible fish but not in resistant animals, which may have some importance for the predisposition to *F. psychrophilum* infection. In contrast, the expression of other regulatory cytokines such as IL2, IL6 (**Figure 4**) and IL17a (not shown) were not significantly different in resistant and susceptible contexts, nor affected by infection. With regards to IFN1, the basal level of expression was slightly higher in resistant fish compared to susceptible ones, and we could observe inductions after bacterial infection. However, this moderate up-regulation was not observed in all individuals.

**Figure 5:**
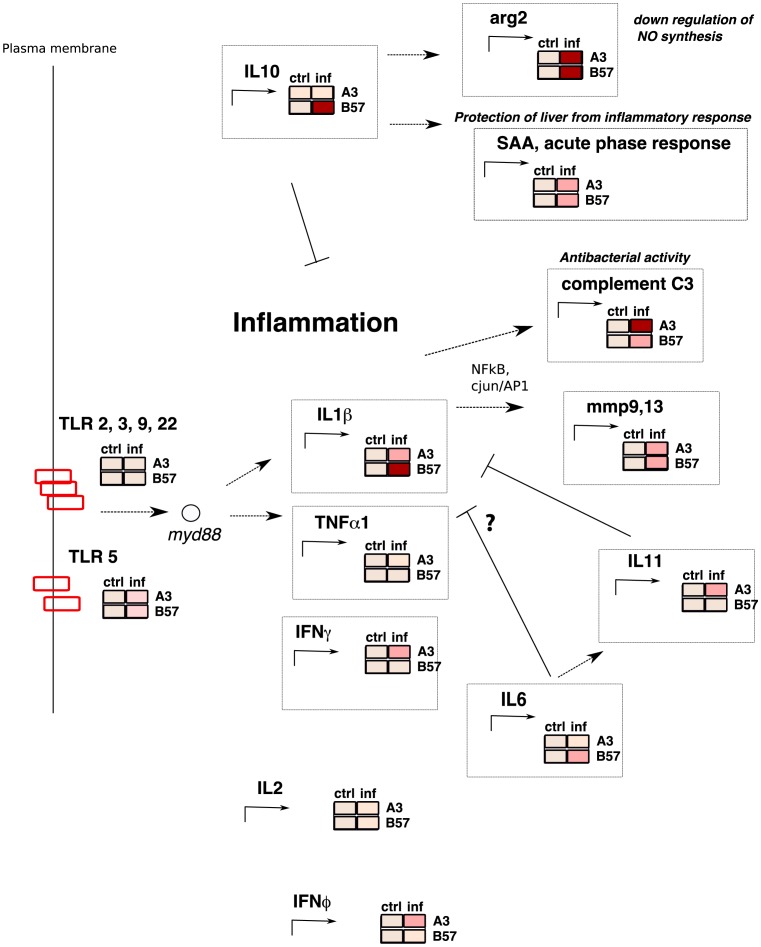
Schematic representation of the impact of *F. psychrophilum* infection on antibacterial immune pathways in clonal fish lines B57_s and A3_r. On day 5 after infection, key pro- (IL1) and anti- (IL10) inflammatory cytokines are up-regulated. While these genes show a greater induction in susceptible fish, which have higher bacterial load, complement C3 is more induced in resistant ones. Data from micro-array and QPCR were aggregated to produce this overview. When available, QPCR ratios were considered. Boxes represent the intensity of induction in red scale (white, no induction; pink, induced with FC>2 only in infected fish and in at least 2 individuals among 3; dark red, induction with FC>5).

These results confirmed and extended the micro-array hybridizations, leading to a tentative view of the perturbations induced by *F. psychrophilum* represented in **Figure 5**. Overall, our results suggest that on day 5 post infection, both pro- and anti- inflammatory genes have been induced in both B57_s and A3_r, but generally at a higher level in the susceptible fish.

### Gene expression differences in the pronephros of uninfected resistant and susceptible fish

The pronephros transcriptome was also compared between control resistant A3_r and susceptible B57_s fish. This analysis identified 812 differential probes (FC>3 or FC<0.33; apv<1%) (**Figure 2B**). Of these 812 probes, only 49 had been identified as differentially expressed between control and infected fish (*i.e.* 6%). GO categories were searched for the whole set of differential probes, and the genes differentially expressed with a GO classification are listed in the **Figure S3**. This set comprised many genes involved in transcription, translation, metabolism (mainly lipid metabolism, which may be especially important for the response to the bacteria), apoptosis and control of cell division, but also genes involved in inflammation, complement activation and antigen processing (n = 26).

In fact, while genes differentially expressed between control resistant and susceptible fish could be involved in the susceptibility to the bacterial infection, the 26 probes that were differentially expressed prior to infection and significantly modulated in infected animals represented only 5% of the whole set of probes modulated in at least one clonal line. Of special interest were two genes reportedly associated to defense mechanisms, a suppressor of cytokine signaling (SOCS) similar to SOCS3 and *IL1r2*, of which basal expression was found higher in the susceptible B57_s fish. In particular, the higher basal expression of the *socs3*-like gene in susceptible fish may be connected to the success of the infection.

## Discussion

We report here the first characterization of the transcriptome response of rainbow trout leukocytes to a systemic bacterial infection by *F. psychrophilum*, a widely distributed pathogen of salmonids and a major problem for aquaculture. Susceptible (B57_s) and resistant (A3_r) clonal lines of rainbow trout were compared to identify pathways determining the genetic predisposition to this bacterial infection.

The primary aim of this study was to produce a global description of the transcriptome modifications induced by *F. psychrophilum* in the pronephric trout leukocytes during the early stages of the infection (5 days pi), when the response is well advanced but has not yet led to major pathology. Since the bacteria were injected i.m. to fish 100–150 g while *F. psychrophilum* typically infects fry and fingerlings, our model likely did not mimic perfectly the natural infection but allowed to identify innate pathways of response to a general infection by *F psychrophilum* in resistant versus susceptible fish. Importantly, transcriptome profiles may vary between different stages of development. Our analysis identified three main sets of genes involved in innate immunity: genes encoding matrix metallo-proteases, genes of pro-inflammatory and regulatory cytokines, and genes encoding anti-bacterial effectors (figure 5). It appeared sometimes difficult to associate a unique trout gene to a given probe. When several probes (each designed from a unique TIGR contig) matched a common counterpart in mammals, they may designate either different genes duplicated in rainbow trout or different splicing isoforms of the same gene. C3 is a good example of this situation, since it is present as multiple copies in the rainbow trout genome [46], which may produce variable signals for the different C3-specific probes present in the array. In such situations, gene-specific expression level could not be easily confirmed by real time QPCR. We therefore considered that the corresponding gene set was induced as a whole, but we could not conclude about the difference of induction of individual genes between resistant and susceptible fish.

As shown by recent studies on *Salmonella typhimurium* and other pathogens in zebrafish [47], we confirm here that transcriptional up-regulation of *mmp9* upon bacterial infections in fish is a general mechanism. *mmp9* has been identified as a target of the TLR5 signaling induced by the flagellin of *S. typhimurium* [47]. In our model, this could not be confirmed since real time QPCR did not show a consistent up-regulation of TLR5, as in zebrafish larvae 24 h after infection by *S. typhimurium*. Thus, our results are consistent with the idea that TLR5 could be complemented by other sensors for the inflammatory response to bacteria [47]. Bacterial induction of another matrix metalloprotease gene, *mmp13*, was also identified by micro-array and confirmed by QPCR. Additionally, a homolog of the metalloprotease Adam8, which is induced by *S.typhimurium* infection in zebrafish [47] and by inflammatory responses in man and in the mouse [48], [49], was up-regulated by *F. psychrophilum* in rainbow trout. Matrix metalloproteinases degrade extracellular matrices, which may have different and opposite impacts on the infection through facilitation of cell migration and bacterial spreading. Interestingly, while *F. psychrophilum* expresses a large diversity of secreted proteases that cause extensive necrotic lesions [38], our results show that it also activates host pro-metalloproteases.

Several other enzymes, which appear to have mainly regulatory/anti-inflammatory functions, have been found up-regulated by the infection. For example, the NADH dehydrogenase 1 alpha subcomplex subunit 5 is part of the mitochondrial respiratory complex and has a regulatory role on inflammation. In the same line, the metallo-reductase STEAP4 down-regulates the production of inflammatory mediators such as IL8 and IL6 in human [50]. Similarly, prostaglandin reductase 1 catalyses an initial step of inactivation of leukotrien-B4. Thus, these results suggest that *F. psychrophilum* induces a strong inflammation that triggers anti-inflammatory pathways in the pronephros.

A number of CC and CXC chemokines were modulated in our micro-array analysis. In the absence of the complete rainbow trout genome sequence, the annotation of chemokines is still preliminary but our results indicate that an important reallocation of immune cells was occurring at the studied stage of the infection. This idea was further supported by *in silico* functional analysis of the micro-array data, since the top network identified by IPA was entitled “Cellular Movement, Hematological System Development and Function, Immune Cell Trafficking”. The pro-inflammatory cytokines IL1β was also clearly up-regulated in the micro-array analysis, which was confirmed by real time QPCR. Among the other tested pro-inflammatory cytokines including TNFα1, TNFα2 and IFNγ, none appeared consistently induced by the infection either in micro-arrays or in QPCR, underlining the primary implication of IL1β in pronephros at this stage of the infection. This observation is consistent with a successive expression of pro-inflammatory cytokines where TNFα2 is highly but early and transiently inducible by the infection. In parallel, genes involved in the IL10 pathway (micro-arrays) as well as IL10 itself (QPCR) were significantly up-regulated by the infection. Real time QPCR also showed that the immuno-regulatory cytokine IL11 was overall induced (Wilcoxon Two Samples Test, *p*≤0.015) though to a lesser extent. These observations suggest that a strong regulatory response had been induced at the time of the analysis. While the functional isoform of IFNΦ1 (also known as type I IFN1) appeared slightly but consistently over-expressed as revealed by real time QPCR, the micro-array experiments clearly showed that the IFN system was not globally activated by *F. psychrophilum* infection. Indeed, while many IFN-induced genes and several Interferon Regulatory Factors (IRF) are well known in rainbow trout and were present in the micro-array, no IRF and only very few IFN–induced genes were found to be modulated. This observation indicated that the infection did not mobilize the IFN system. Other cytokines such as IL2, IL6, and IL17D showed no significant modification of expression upon *F. psychrophilum* infection in the context analyzed herein. In contrast, the complement cascade as well as AMP and acute phase response genes were significantly modulated by the infection (FC>3), confirming their importance in the host reaction.

The second aim of this study was the identification of differences between the transcriptome responses of resistant and susceptible fish. Our micro-array analysis revealed differences between transcriptomes of the resistant A3_r and susceptible B57_s fish before infection. However, these differences did not involve key genes or pathways obviously connected to the antibacterial response or to inflammation, and rather suggested significant differences in metabolism and other basic processes. Hence, the understanding of their role in the susceptibility to the disease will require combined genetic and functional approaches. Also, the kinetics of the bacterial load, as well as the kinetics of the transcriptome modifications induced by *F. psychrophilum*, would have to be characterized to produce a comprehensive comparison of the respective responses of the two clonal lines. In fact, the difference of bacterial loads in the pronephros suggests that the bacteria escape immune control in the susceptible line.

Still, several differences in the up-regulation pattern 5 days after infection deserve to be noted, as shown on figure 5.

A number of genes involved in antibacterial immunity (including complement C3) were induced with a greater magnitude in resistant compared to susceptible fish. C3 plays a key role in the activation of the complement cascade, and its cleavage is necessary for both classical and alternative pathways. Its critical importance is well illustrated by the higher susceptibility of C3 deficient humans and mice to bacterial infections [51], [52]. In a few cases, a greater up-regulation of a given gene in resistant fish did not lead to higher final expression level. Thus, several genes including lysozyme CII and SAA had a basic expression level lower in the resistant clonal lines, and reached after infection a final level equal or lower in resistant compared to susceptible fish. While expression difference on day 5 post-infection may represent a delayed response due to slower bacterial growth in resistant fish, these genes represent interesting candidates to explain the higher resistance in our fish clonal line.

In contrast, several key genes of inflammation were up-regulated much more in infected susceptible animals, where a higher bacterial load was measured. Since these genes were expressed at comparable basal levels in naive fish from both clonal lines, such inductions really led to higher expression levels in susceptible fish. An important example was the pro-inflammatory cytokine IL1β, and the gene *Il1r2* encoding the IL1 receptor was similarly induced. Interestingly, the expression of the anti-inflammatory cytokine IL10 and, to a lesser extent, IL11 mirrored IL1β. Further work will be necessary to characterize the kinetics of IL10 expression in susceptible and resistant fish. Indeed, IL10 has been recently identified as a critical modulatory factor of innate immunity in a mouse model of *S. typhimurium* infection [53]. IL10 is also a negative regulator of phagocytosis and its expression may impact significantly phagocyte infection and resulting intracellular growth as described for *F. psychrophilum* in [54]. When examining micro-array data in search of genes involved in phagocytosis, we could not identify clear trends after infection, but we found four sequences potentially involved in this process, of which 3 where more expressed in resistant than susceptible naive fish: ELMO1 (TC109245), COR1A (TC 100726 & 120552), CD209 (TC130714) and ABCA1 (TC 125779). On the other hand, IL10 expression is critical for the protection of liver of the host against the inflammatory response, and the high expression observed in susceptible fish with a high bacterial load could be simply linked to this activity.

In conclusion, our results show that infection by *F. psychrophilum* induces a robust inflammatory response in both resistant and susceptible fish clonal lines, as shown in figure 5. While a common core response is observed in both lines, differences have been observed that might be involved in the resistance to *F. psychrophilum* infection. This first analysis of the leukocyte transcriptome in susceptible and resistant fish following *F. psychrophilum* infection paves the way for a combined functional and genetic characterization of the resistance to this bacterium.

## Supporting Information

Figure S1 **This excel file contains the complete list of up- and down- regulated genes in rainbow trout clonal lines B57_s and A3_r following** ***F. psychrophilum*** **JIP 02/86 infection.** Probes have been classified by fold change and adjusted p value.
(XLSX)

Figure S2 **This table contains the list of probes for which up- or down- regulation was significant in only one of the two fish clonal lines, while a high adj.** ***p*** **value in the other line indicated a large variation of the expression level.**
(XLSX)

Figure S3 **This table contains the list of genes differentially expressed between the two fish clonal lines prior infection.** GO categories were identified for the whole set of probes.
(XLSX)
